# SERS spectroscopy with machine learning to analyze human plasma derived sEVs for coronary artery disease diagnosis and prognosis

**DOI:** 10.1002/btm2.10420

**Published:** 2022-10-05

**Authors:** Xi Huang, Bo Liu, Shenghan Guo, Weihong Guo, Ke Liao, Guoku Hu, Wen Shi, Mitchell Kuss, Michael J. Duryee, Daniel R. Anderson, Yongfeng Lu, Bin Duan

**Affiliations:** ^1^ Department of Electrical and Computer Engineering University of Nebraska Lincoln Lincoln Nebraska USA; ^2^ Mary & Dick Holland Regenerative Medicine Program University of Nebraska Medical Center Omaha Nebraska USA; ^3^ Division of Cardiovascular Medicine, Department of Internal Medicine University of Nebraska Medical Center Omaha Nebraska USA; ^4^ Department of Industrial and Systems Engineering Rutgers, The State University of New Jersey Piscataway New Jersey USA; ^5^ School of Manufacturing Systems and Networks Arizona State University Mesa Arizona USA; ^6^ Department of Pharmacology and Experimental Neuroscience University of Nebraska Medical Center Omaha Nebraska USA; ^7^ Division of Rheumatology, Department of Internal Medicine University of Nebraska Medical Center Omaha Nebraska USA; ^8^ Department of Surgery, College of Medicine University of Nebraska Medical Center Omaha Nebraska USA; ^9^ Department of Mechanical and Materials Engineering University of Nebraska‐Lincoln Lincoln Nebraska USA

**Keywords:** coronary artery disease, diagnostics, machine learning, small extracellular vesicles, spectrogram

## Abstract

Coronary artery disease (CAD) is one of the major cardiovascular diseases and represents the leading causes of global mortality. Developing new diagnostic and therapeutic approaches for CAD treatment are critically needed, especially for an early accurate CAD detection and further timely intervention. In this study, we successfully isolated human plasma small extracellular vesicles (sEVs) from four stages of CAD patients, that is, healthy control, stable plaque, non‐ST‐elevation myocardial infarction, and ST‐elevation myocardial infarction. Surface‐enhanced Raman scattering (SERS) measurement in conjunction with five machine learning approaches, including Quadratic Discriminant Analysis, Support Vector Machine (SVM), K‐Nearest Neighbor, Artificial Neural network, were then applied for the classification and prediction of the sEV samples. Among these five approaches, the overall accuracy of SVM shows the best predication results on both early CAD detection (86.4%) and overall prediction (92.3%). SVM also possesses the highest sensitivity (97.69%) and specificity (95.7%). Thus, our study demonstrates a promising strategy for noninvasive, safe, and high accurate diagnosis for CAD early detection.

## 1 INTRODUCTION

Coronary artery disease (CAD) is represented by the accumulation of atheromatous plaques within the walls of the arteries that supply blood to the heart.^1^ CAD is one of the major cardiovascular diseases and remains the leading causes of death worldwide.^2^, ^3^, ^4^ CAD is responsible for about 7 million deaths worldwide.^5^, ^6^ Based on the degree of stenosis and plaque characteristics, CAD patients can present clinically with cardiac symptoms and can be divided into different categories, that is, patients with stable nonobstructive plaques (SP), non‐ST‐elevation myocardial infarction (NSTEMI) and ST‐elevation myocardial infarction (STEMI). This reflects the continuum of CAD and with increased severity there are decreased lumen areas, greater plaque burden, more plaque rupture all of which are associated with a greater risk of mortality.^1^, ^7^ Thus, there is still a huge need to develop new diagnostic and therapeutic approaches for CAD treatment, especially an early accurate CAD detection and timely intervention, which is expected to avert many late CAD events and deaths.

In the past few decades, various preventive and therapeutic strategies have substantially improved the prognosis of patients suffering from CAD. Many advanced techniques have been developed, reported, and clinically applied to the diagnostic and prognostic workup of CAD, such as electrocardiogram,^8^, ^9^ echocardiography,^10^ intravascular imaging,^11^, ^12^ coronary angiography.^13^, ^14^ Although these diagnostic methods have revolutionized the management of CAD patients, the prevalence of adverse cardiac events remains high. Imaging approaches, like coronary angiography, are invasive and should not be used to diagnose the early‐stage CAD. Some chemical agents used in cardiac stress testing, such as radiocontrast media may have potential side effects for CAD patients. As well, these stress tests are designed to detect significant CAD which represents a luminal loss of more than 50%. Novel biomarkers can predict and differentiate between CAD types in both the early and late stages, and may reduce unnecessary invasive coronary angiography and thus enhance predictive value.^15^ However, the detection sensitivity of these biomarkers has been lower due to the use of large amounts of capturing and labeled detecting antibodies, which may increase the risk of false positivity due to nonspecific binding with nontarget analytes, especially for early CAD stage.^16^, ^17^ In addition, patients may not have any symptoms in the early CAD stage reflective of nonobstructive CAD stage (SP stage). Therefore, the prompt, economical, and accurate low‐risk diagnosis and prognosis for CAD in asymptomatic patients are crucial to allow timely prevention and therapeutic treatments to improve patients' quality of life prior to the event. Enhancing the early diagnosis of CAD and utilization of therapeutic approaches to prevent progression is crucial for the management and prevention of CAD.

Small Extracellular vesicles (sEVs) are small lipid‐bilayer enveloped assemblies with sizes ranging from 20 nm to several micrometers.^18^, ^19^ sEVs are secreted by all cells in both normal and diseased tissues, and can be further categorized based on their biogenesis, size, and biophysical properties, such as exosomes, apoptotic bodies, microvesicles, ectosomes, and other vesicles.^20^, ^21^, ^22^, ^23^ sEVs are found in most biological fluids and contain a wide variety of cargo, such as proteins, lipids, nucleic acids and metabolites.^21^, ^24^, ^25^ These cargoes are representative of their cellular origin and reflective of the pathological condition of the origin tissue and cells, which may serve as noninvasive diagnostic biomarkers in biological fluids.^26^, ^27^, ^28^ Numerous studies have reported the value of exploiting sEV in diagnostic and therapeutic applications in the central nervous,^29^, ^30^ cancer,^31^, ^32^ and visceral organ diseases.^33^, ^34^ The cargoes of these sEVs reflect the molecular content and pathology of their original cells.^23^ Therefore, sEVs isolated from liquid and tissue biopsies can serve as potential biomarkers to follow disease progression.^35^, ^36^, ^37^, ^38^, ^39^ Different diseases are expected to alter either the sEV contents or the sorting and packaging process^37^, ^39^ and these alterations are expected to be detectable and be useful in diagnosis for evaluating disease activity and/or the response to therapy.^40^ Plasma as an important component of blood, has the characteristics of representing systemic disease pathology.^41^ In the cardiovascular system, sEVs are associated with endothelial cells, cardiac myocytes, vascular cells, progenitor and stem cells, and play an essential role in the development, injury and disease of the cardiovascular system.^28^, ^42^ Thus, cardiovascular‐derived plasma sEVs have great potential as potential diagnostic biomarkers for CAD screening.

Surface‐enhanced Raman scattering (SERS) is a commonly used sensing technique in which inelastic light scattering by molecules is greatly enhanced when the molecules are absorbed onto corrugated metal surfaces (usually Au).^43^, ^44^ The label‐free, nondestructive and noninvasive characteristics of SERS enable its biomedical application to the diagnosis of diseases, such as neurodegenerative disorders,^45^ cancer,^46^, ^47^ or diabetes.^48^ This innovative technique has also been used to diagnose lung cancer by combing with exosomes by pattern analysis of SERS data.^49^, ^50^ Thus, SERS has the potential to differentiate sEVs based on their different membrane lipid/protein contents along with other various functional groups. However, the Raman signatures of sEVs are expected to be highly complex due to the overlapping. The common solution is to analyze the entire Raman spectra as “fingerprint” input by leveraging the power of machine learning (ML). The sEVs collected from patients with different stages of CAD have “impact” on the entire Raman spectra (spectral shapes), although the changes are usually small and very difficult to be detected. By ML algorithms and large training data set, it is possible to detect common “patterns” from the hundreds of Raman spectra with training data and then the algorithm can perform prediction in blind tests. ML has been extensively applied in analyzing spectroscopic signals for complex bio‐samples and has achieved satisfying results. Thus, by using ML assisted analysis on a high‐dimensional SERS database, valuable information is expected to be extracted for accurate estimation and practical prediction of known CAD stages which when validated can be used clinically as a diagnostic tool.

**FIGURE 1:**
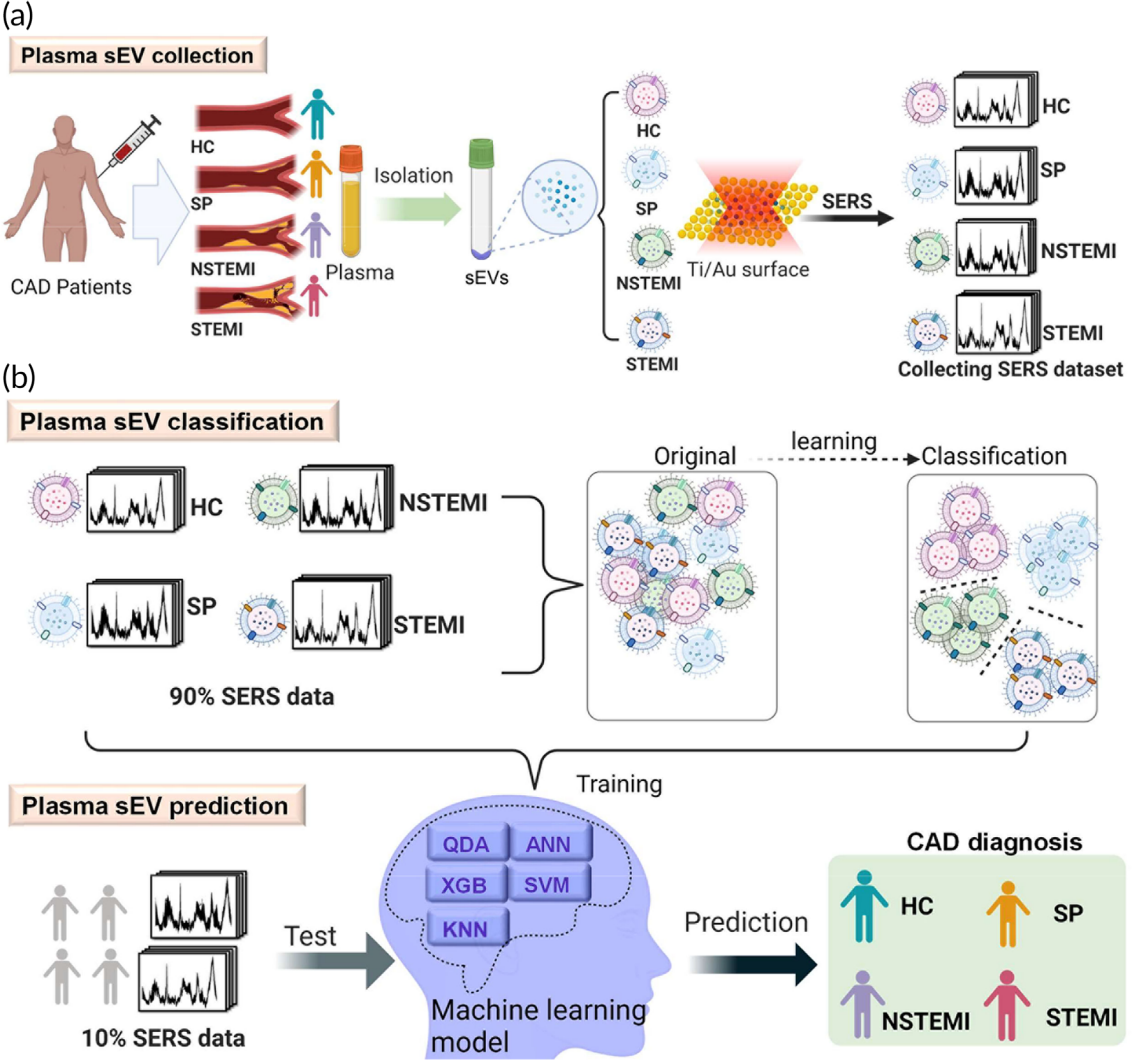
Schematic illustration of ML‐assisted sEV analysis for CAD diagnosis. (a) Isolation of sEVs from the human plasma of patients with different CAD stages, including HC, SP, NSTEMI and STEMI stages, and collection of spectroscopic data of plasma‐derived sEVs by SERS. (b) Overview of ML‐assisted plasma sEV classification and CAD diagnosis using sEVs SERS signal patterns. This illustration is created by BioRender.com with an authorized license

In this study, we demonstrated a noninvasive, label‐free SERS technique to diagnose CAD by assessing and monitor progression of the disease. To the best of our knowledge, direct, label‐free in vitro characterization of sEVs for the early CAD stage diagnosis through SERS at a biomolecular level has not previously been demonstrated. A Raman spectral library of plasma‐derived sEVs from patients with various degrees of CAD, including SP, NSTEMI, and STEMI CAD stages, was developed. Plasma from patients without CAD was used as healthy control (HC) group. We hypothesized that SERS data combining with ML algorithms can accurately classify the sEVs from different CAD stages and be used to predict potential risks in CAD patients. Firstly, we isolated and characterized sEVs from human plasma samples with various degrees of CAD and collected their SERS signals (Figure 1a). Then, the SERS spectra of plasma‐derived sEVs were obtained by Raman microscopy and analyzed using five supervised ML models (Figure 1b), including Quadratic Discriminant Analysis (QDA), Support Vector Machine (SVM), K‐Nearest Neighbor (KNN), Artificial Neural network (ANN), and XGBoost (XGB). Then, 90% of SERS data set of sEVs were used to train the models. The supervised models classified the sEVs data into four clusters (one HC group and three stages of CAD). The remaining 10% spectral data were used to predict their CAD stages through the models. The five methods used in this study are most common algorithms in ML for classification and prediction. Each method represents a typical type of algorithms in ML theory. All methods have predictive errors and statistical noises in the data, especially for large data set or data set with sampling limitations. Therefore, it is important to understand the performance difference among these methods. Thus, we compared the diagnosis performances of the five models and demonstrated robust classifications and high accurate diagnosis in plasma‐derived sEVs for early‐stage CAD detection and CAD progression monitoring through the introduction of ML algorithms.

## 2 RESULTS AND DISCUSSION

### 2.1 Plasma‐derived sEV isolation and characterizations

**FIGURE 2:**
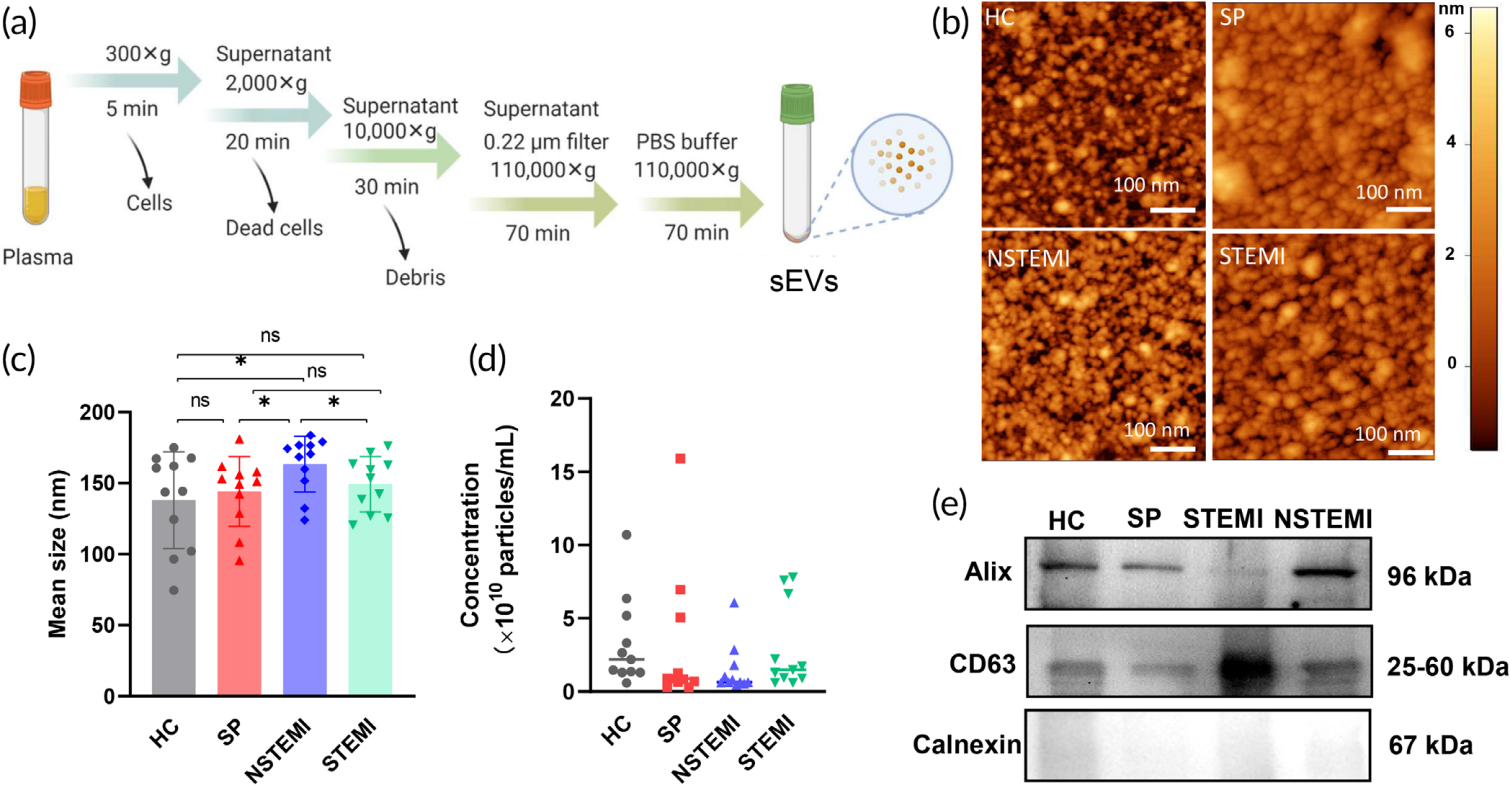
Isolation and characterizations of sEV from human plasma. (a) Scheme of isolation procedure of sEVs from human plasma. (b) AFM images of sEVs. (c, d) The size and concentration of sEVs by NTA test (*n* = 11, *p < 0.05). (e) Western blot analysis of biomarker proteins on sEVs

Plasma‐derived sEVs were isolated from patient plasma using a standard procedure of ultracentrifugation as shown in Figure 2a. Ten samples from each group were used to evaluate plasma‐derived sEV features. The AFM images showed that the vesicle morphology of the most vesicles was flattened sphere‐like with nanoscale sizes (Figure 2b), which was consistent with previous results.^51^ The vesicles typically consisted of membrane vesicles of 50–200 nm in diameter according to the results of nanoparticle tracking analysis (NTA) (Figure 2c,d). The sEVs in the HC group had relatively smaller average size (Figure 2c). Additionally, the average size of vesicles in NSTEMI group was the largest among the four groups with significant difference (Figure 2c). The ranges of sEV concentration had wide distribution, and there was no significant difference among different groups (Figure 2d). The markers of sEVs, the tetraspanin (CD63) and endosomal pathway protein (Alix) were detected by Western blotting (Figure 2e). Both Alix and CD63 were expressed in sEVs and Calnexin (negative marker) was not detected. These results confirmed that the recovered vesicles were sEVs. Taken together, the size and content of typical sEV protein markers indicated that sEVs were successfully isolated from the human plasma samples from different stages of CAD patients.

### 2.2 Averaged Raman spectra for four stages of CAD and QDA classification

**FIGURE 3:**
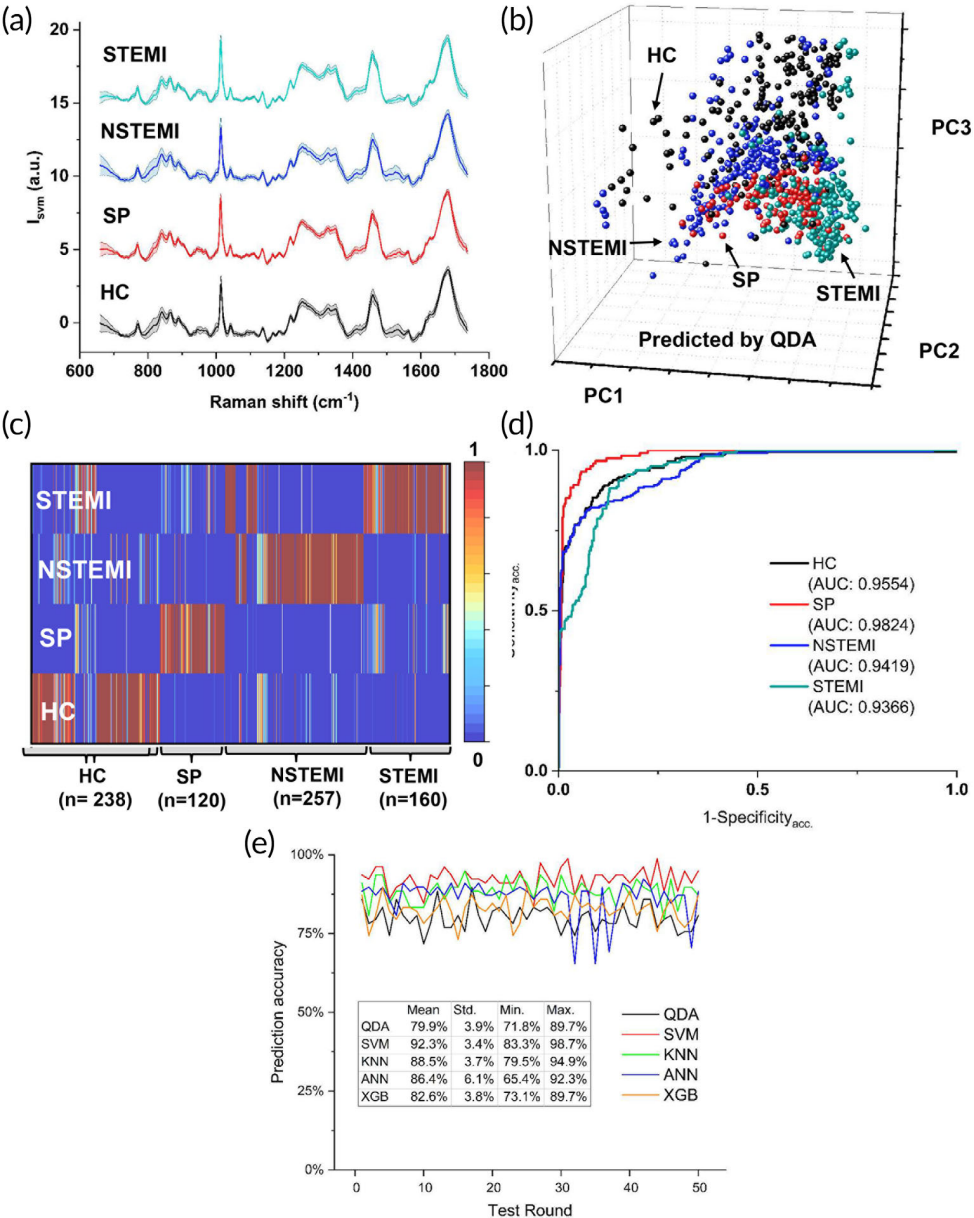
Raman spectra and QDA analysis. (a) Averaged Raman spectra for four stages of CAD (HC, SP, NSTEMI, STEMI); (b) The PCA plot colored by QDA classification results of the total 775 Raman spectra from four stages of CAD. (c) Heat map of the prediction possibility (range 0–1) by QDA for four stages of CAD. (d) ROC curves for each representation stage and their AUC values. (e) Prediction accuracy of test sets performed in QDA, SVM, KNN, ANN, and XGB in 50 test rounds by randomly splitting 90% of data into a training set and 10% into a test set

To provide an overview of the Raman spectra of four CAD stages, all spectra after standard normal variate processing were simply averaged and shown in Figure 3a. In the fingerprint region, spectra showed Raman peaks appeared to originate from lipids and proteins which are the major contributors to sEV surface. For example, the vibrations contributed to symmetric ring breathing of tryptophan appeared as a peak at 755 cm^−1^. A peak at 830 cm^−1^ was observed corresponding to C—O—O vibration typical of phospholipids. Other peaks such as 856 cm^−1^ (glycogen), 879 cm^−1^ (C—C stretch proline ring), 1005 cm^−1^ (phenylalanine), 1126 cm^−1^ (C—C vibrations in lipid), 1244 cm^−1^ (amide III), 1450 cm^−1^ (CH_2_ bending vibration of proteins/lipids), and 1668 cm^−1^ (amide I) were also observed.

When we closely look at each individual spectrum as shown in Figure S1, the variations in spectra were significantly notable not only across the four CAD stages but even within the same CAD stage. The large variations are expected, because in spectroscopic measurement, the obtained data is semiquantitative, which means that analyzing only based on a single pathology‐related Raman spectroscopic peak is unlikely to be reliable and not suitable for diagnosis or classification of the disease status. The solution for better understanding of the data is to apply ML methods to extract the diagnosis or classification information based on the entire spectral pattern.

The first method we applied is discrimination analysis, which has been widely used to classify and predict Raman spectra of various biological and biomedical samples.^52^ In this study, we chose QDA method since the classification (decision) boundaries of QDA can be learned quadratically and flexibly compared to another often used method, linear discrimination analysis (LDA). The PCA plot colored by QDA classification results is shown in Figure 3b, where each colored dot represents a spectrum. It clearly shows the capabilities to separate different sEV subpopulations based on the SERS spectra; however, overlaps are still observed. The overall classification accuracy performed by cross‐validation is 80.26% with a sensitivity of 96.28% and a specificity of 74.37%. The sensitivity and specificity were calculated by counting HC group as negative and other three CAD stages together as positive. The heat map (Figure 3c) summarizes the prediction possibility (range 0–1) by QDA for the HC group and three stages of CAD. Red color indicates the highest possibility (1), while blue color indicates the lowest possibility (0). By supervised QDA, the heat map shows most predictions fall in the correct stages, but mispredictions and swings between two stages (yellow color, ~0.5) are still observed. To draw each receiver operating characteristic (ROC) curve, we counted the representation stage as a positive response, and the other three stages as the negative response for the calculation of Sensitivity_acc_ and Specificity_acc_. As an example, in the ROC curve of HC, the Sensitivity_acc_ and Specificity_acc_ are calculated as results of HC response against other three stages (SP, NSTEMI, and STEMI) responses. Area under the curve (AUC) of ROC is the indicator of the goodness of fit for the model, and a value of 1 indicates a perfect fit and a value near 0.5 indicates that the model cannot discriminate among the stages. The AUC results by QDA for each stage range from 0.9366 to 0.9824, indicating the QDA method shows good fitting results (Figure 3d). Furthermore, due to possible overfitting by the cross‐validation method, we also verified the QDA model by randomly splitting data into training and testing sets. In each round, 90% of data (total 697 spectra) was used as a training set, and the remaining 10% of data (total 78 spectra) was used as a testing set. Only the training set was used to train the model, and the testing set was used for blind prediction. A total of 50 rounds were performed to evaluate the loss and accuracy change of the testing set. As shown in Figure 3e, we found the averaged overall accuracy of QDA is 79.9% ± 3.9%, with a minimum of 71.8% at round 10th and a maximum of 89.7% at round 12th, which is consistent with the cross‐validation results.

### 2.3 KNN, ANN, SVM, and XGB classifications and predictions

Besides QDA, we also implemented four other ML algorithms, including KNN, ANN, SVM, and XGB, to classify and predict CAD stages and compare the classification performances. Each method has its own advantages and weaknesses.^53^ QDA method allows nonlinear fitting through data and the relationship between data and data interpretation could be established practically. KNN method depends on the nearby adjacent samples rather than the algorithm of discriminating the class domain for classification, which may be more suitable for dense overlapped data sets among multiple classes. ANN has more tolerance to noise and missing data, and is good at handling high‐dimensional data sets, but may have difficulty with data interpretation and algorithm structure understanding. SVM is also durable to noise and has advantage in handling a small set of data and overfitting issues. XGB is fast, but ideally requires nonoverlapped data set and an inappropriate training set may lead to distorting the decisions.

Same as QDA, the raw Raman data was preprocessed and performed by PCA initially. Fifteen PCs were chosen (Figure S2) as the new input variables to the models. Then to compare the classification accuracies from different methods, the model parameters also need to be estimated properly since those parameters will significantly affect the efficiency of the classification. Thus, obtaining the best possible set of parameters is critical, both from the computing cost and computing accuracy. Among these four classification methods, all of them have parameters that need to be determined. For ANN, the input is 15 PCs, and the output is the 4‐classification group. To avoid overfitting or underfitting, an ANN structure with two hidden layers was chosen. The first hidden layer was set to have 10 neurons, and the second hidden layer was set to have 6 neurons. For XGB, 5000 decision trees were chosen. For SVM, the radial basis function kernel was chosen. In KNN, the best accuracy was obtained when *K* = 1 after experimenting *K* = 1–30. For all methods, 90% of the full data set was randomly split for training while the remaining 10% was used for testing. The process was also repeated for 50 rounds. Figure 3e shows the prediction accuracies for all the five classification methods used in this study. Results demonstrated that the SVM provided the highest and most robust prediction accuracy of 92.3% with a SD of 3.4% among all five methods. XGB provides a low prediction accuracy of 82.6%, but still better than previously used QDA method (79.9%). Among the 50 round tests of ANN, although the averaged prediction accuracy of ANN is 86.4%, there were 3 rounds of prediction accuracy below 70%, indicating ANN may lack stability due to a randomly chosen training set. The averaged confusion matrix from the 50 round tests is also shown in Table S1. For early CAD prediction (SP stage), QDA, SVM, and ANN had the prediction accuracy of 88.7%, 85.9%, and 86.4%, respectively, much higher than the other two methods, which indicates these ML algorithms may work better for early CAD detection. For the most severe stage (STEMI) prediction, all five algorithms have similar performance. QDA, SVM, KNN, ANN, and XGB had the prediction accuracy of 84.7%, 88.5%, 86.8%, 89.1%, and 84.2%, respectively. For HC group prediction, SVM and KNN have higher prediction accuracies of 95.7% and 91.6% over other three algorithms. Through Table S2, we found SVM has the overall best performance over other four algorithms for prediction of all stages. Table 1 shows the averaged confusion matrix of sensitivity and specificity from the 50 round tests. SVM method also had the highest sensitivity (97.2%) and specificity (95.8%) among all the methods used in this study, since major classification errors occurred within the disease groups (SP to be misclassified as NSTEMI and NSTEMI to be misclassified as SP). QDA shows the lowest specificity (74.8%) among the five methods.

**TABLE 1:** Averaged confusion matrix of sensitivity and specificity from the 50 round tests

			Predicted class			
Model	Sample comparison		Positive	Negative	Sensitivity	Specificity
QDA	SP + NSTEMI + STEMI vs HC	Positive	51.8	1.9	96.5%	74.8%
		Negative	6.0	17.8		
SVM	SP + NSTEMI + STEMI vs HC	Positive	52.2	1.5	97.2%	95.8%
		Negative	1.0	22.8		
KNN	SP + NSTEMI + STEMI vs HC	Positive	50.5	3.2	94.0%	91.6%
		Negative	2.0	21.8		
ANN	SP + NSTEMI + STEMI vs HC	Positive	51.0	2.7	95.0%	88.7%
		Negative	2.7	21.1		
XGB	SP + NSTEMI + STEMI vs HC	Positive	49.0	4.7	91.2%	82.4%
		Negative	4.2	19.6		

### 2.4 SVM as the best ML method in this study

**FIGURE 4:**
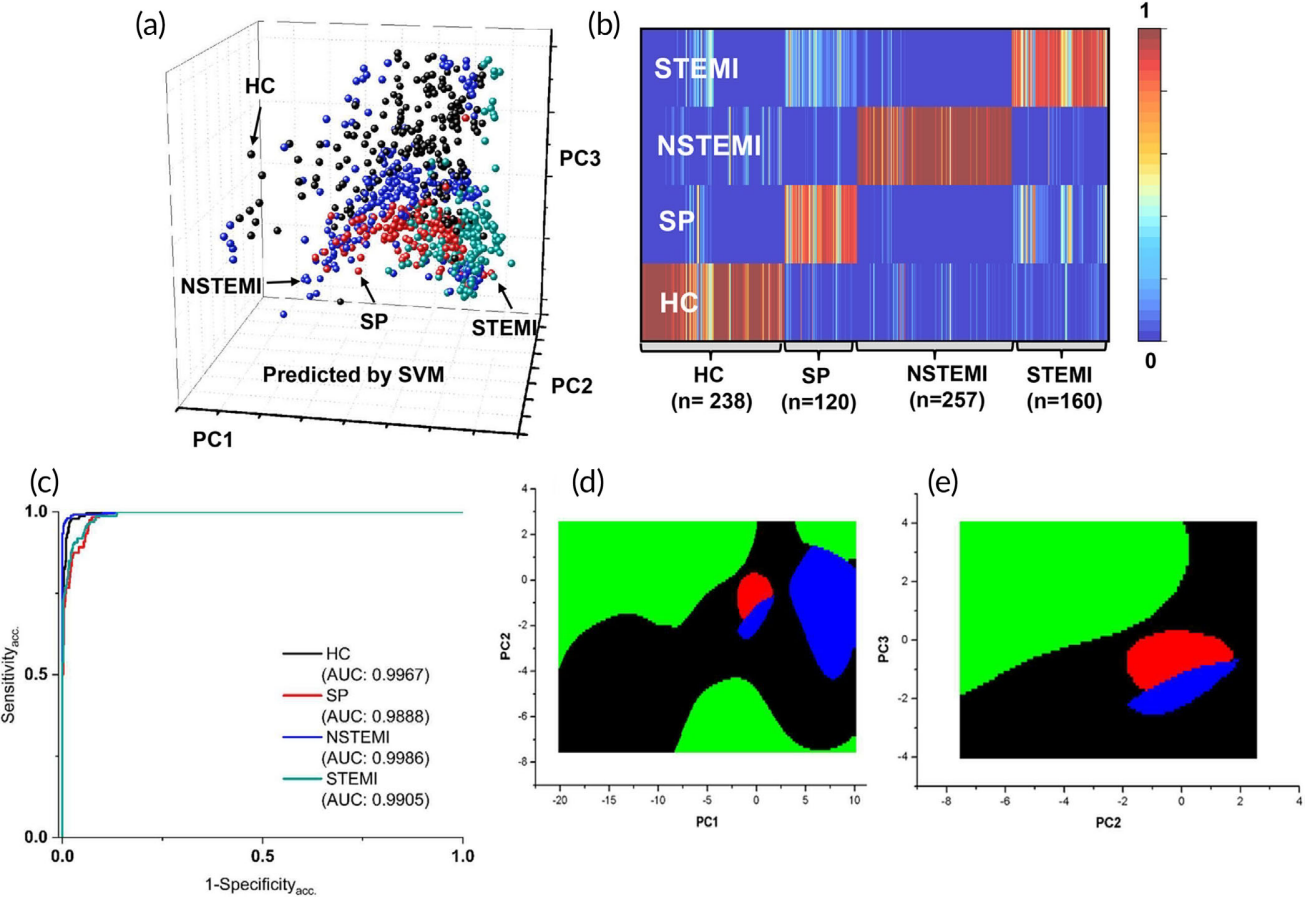
SVM analysis. (a) A PCA plot colored by SVM classification results of the total 775 Raman spectra from four stages of CAD. (b) Heat map of the prediction possibility (range 0–1) by SVM for four stages of CAD. (c) ROC curves for each representation stage and their AUC values. (d) Decision boundaries of SVM projected in 2D PCA plot (PC1 vs PC2). (e) Decision boundaries of SVM projected in the 2D PCA plot (PC2 vs PC3). (Black: HC; Red: SP; Green: STEMI; Blue: NSTEMI)

The PCA plot colored by SVM classification results is shown in Figure 4a, where each colored dot represents a spectrum. The heat map (Figure 4b) summarizes the prediction possibility (range 0–1) by SVM for the HC group and three stages of CAD. Red color indicates the highest possibility (1) while the blue color indicates the lowest possibility (0). The heat map shows almost all predictions fall in the correct stages with a few swings between two stages (yellow color, ~0.5). The AUC results by SVM for each stage range from 0.9888 to 0.9967, indicating the SVM method shows excellent fitting results in Figure 4c, Figure 4d,e shows the decision boundaries of SVM projected in the 2D PCA plot, PC1 vs PC2, and PC2 vs PC3, respectively.

### 2.5 Challenges and future directions

sEVs serve as a mediator of intercellular communication between cells, and can be used as a noninvasive indicator of disease,^54^, ^55^, ^56^ which is the strategy applied in this study to diagnose CAD status via detecting and analyzing SERS signals from sEVs. However, although as a promising clinical approach, the application of our study still faces several challenges. The first limitation is related to the major Raman signals that are mostly derived from sEV surface molecules (e.g., membrane proteins, lipids).^49^, ^57^, ^58^ Thus, SERS signals may be influenced by various membrane molecules.^59^ In order to identify protein signatures existing in both surface and interior of sEV, we will analyze sEV cargoes using other advanced technologies such as proteomics.^60^ The second challenge is the patient number involved in this study. One solution is to expand the obtained Raman library; however, another issue may come out that the measurements between different batches of patient samples obtained from national wide may vary largely due to uncertain uniformity among different SERS methods and system errors coming from different Raman instruments.^44^ Thus, it has to optimize the calibration condition before each test to improve the precision of detection and analysis. The third challenge is that the isolated plasma sEVs may contain a variety of sEVs derived from other organs and cells that are not related to the CAD.^34^, ^61^ Therefore, our future study will focus on expanding the sample number and types of sEV samples to improve the robustness and reliability of our approach, such as detection of sEVs from heart tissues with different CAD stages.

## 3 CONCLUSIONS

In summary, we successfully isolated sEVs from the human plasma samples from four stages of CAD patients, that is, HC, SP, NSTEMI, and STEMI. SERS measurements in conjunction with five ML algorithms were then applied for the classification and prediction of the sEV samples. The overall accuracy was 79.9%, 92.3%, 88.5%, 86.4%, and 82.6% for QDA, SVM, KNN, ANN, and XGB, respectively. Among these five approaches, SVM shows the best prediction results on both early CAD detection (86.4%) and overall prediction (92.3%). SVM also possesses the highest sensitivity (97.69%) and specificity (95.7%). Thus, our study demonstrates a promising strategy for noninvasive, safe, and high accurate diagnosis for early CAD detection.

## 4 MATERIALS AND METHODS

### 4.1 sEV isolation and characterizations

Deidentified human plasma samples were donated by the Nebraska Cardiovascular Biobank and Registry (IRB approved protocol 133‐14‐EP). The human plasma was obtained from patients at the time of cardiac catheterization and immediately stored at −80°C for further use. Samples were obtained from patients who presented with chest pain and a positive stress test, and those who presented with a NSTEMI or a STEMI. Patients who had nonobstructive CAD (i.e., <50% lesions) at catheterization were defined as stable CAD patients (SP). The bank ID, Gender, Age, Race of each patient is recorded in Table S2. sEVs were isolated from patient plasma with three CAD stages, and the number of patients is 15 (SP stage), 15 (NSTEMI stage), and 17 (STEMI stage), respectively. The control samples (HC stage) were from 17 patients that had no signs of CAD. Among all sEV samples, 13 HC samples, 6 SP samples, 13 NSTEMI samples, and 8 STEMI samples were chosen for SERS test, and other samples not used in SERS test were applied for NTA. The human plasma was first centrifuged at 300 ×*g* for 5 min, 1000 × *g* for 20 min and then at 10,000 × *g* for 30 min sequentially. The supernatant was then filtered with a 0.22 μm filer, and ultracentrifuged (Sorval X + 80 Ultracentrifuge, Thermo Fisher) at 100,000 *g* for 70 min. Subsequently, the sEV pellet was washed with PBS and ultracentrifuged at 100,000 × g for 70 min. The collected sEVs were reconstituted in PBS buffer and then preserved at −80°C. For SERS analysis, parts of exosomes were resuspended in phosphate buffer (PB) (0.1 M, pH = 7.4). The size and concentration of the final sEVs were examined by NTA using a NanoSight (NS300) measurement. The exosome morphology was evaluated as previously described by using atomic force microscopy (AFM, Bruker).^62^

### 4.2 Western blot

The markers (CD63 and Alix) of the isolated sEVs were correspondingly detected by Western blotting as reported previously.^62^, ^63^ The sEVs were lysed using the Mammalian Cell Lysis kit (Sigma–Aldrich) and quantified using Pierce™ BCA Protein Assay Kit (Thermo Fisher Scientific, 23227). The samples were preheated at 60°C for 15 min. Proteins were electrophoresed in an SDS‐polyacrylamide gel followed by transferring to PVDF membranes. The membranes were blocked with 5% BSA in TBS‐Tween 20 and were then probed with antibodies specific for CD63 (1:1000, ab216130, Abcam), Alix antibody (1:1000, Proteintech), and calnexin (1:1000, Proteintech, negative biomarker for sEVs) overnight at 4°C. After three washes in TBS‐tween 20, membranes were incubated with the secondary antibody (Thermo Scientific) for 1 h and washed again. For visualization, blots were exposed to SuperSignal West Dura Extended Duration substrate and measured by the FluorChem R system (ProteinSimple).

### 4.3 SERS measurements

The sEV samples were immediately measured within 12 hours after taking from −80°C. Gold‐coated glass slides (Ti/Au 40 nm/100 nm, Deposition Research Lab Inc.) were used for better suppression of fluorescence background and surface plasmonic enhancement for SERS. Five microliters of each sEV samples was dropped onto the gold slide and then measured immediately before drying at room temperature. Raman spectra were recorded using a commercial micro‐Raman microscope (Renishaw InVia Reflection) with 633‐nm diode laser excitation. The laser power was set to 10 mW. The laser beam was focused on the 5 μl droplet by using a 50× microscope objective with a numerical aperture of 0.75 (Leica n PLAN EPI 50×/0.75). The laser spot is estimated to be 1 μm in diameter. In the experiment, due to the sample availability, the sample size was experimentally boosted by automated measuring at different spots of the sEV droplet in 4 × 5 grid. Each Raman spectrum obtained for following analyzes was recorded with an exposure time of 1 s and accumulated by 10 times. The Raman data set is shown in Figure S3. Since the measurement was automated and conducted on the sEV droplets by mapping program, spectra with high background levels (high PBS peak and high background level) were obtained and then should be removed from the data set (Figure S4) to minizine the buffer influence on the following machine learning analysis. Thus, the total number of Raman spectra obtained is 775, including 238 from 13 HC samples, 120 from 6 SP samples, 257 from 13 NSTEMI samples, and 160 from 8 STEMI samples.

### 4.4 Data preprocessing and classification methods

Data preprocessing of the raw Raman spectra included baseline correction and normalization. Baseline correction was performed by Vancouver Raman algorithm with five‐point boxcar smoothing and a five‐order polynomial fit. After baseline correction, the spectra were normalized using the standard normal variate technique, which can remove multiplicative error and preserve each preprocessed spectrum having same contribution to the following classification analysis.

### 4.5 ML and classification methods

Classification analysis was adopted to identify the CAD stage based on SERS measurements. Raman spectra were processed to reduce dimensionality, and then input to classifiers for CAD stage prediction. Cross‐validation (CV) was adopted for robust model training and validation. The diagnostic capability of the classification models, characterized by sensitivity and specificity, was analyzed with Receiver Operating Characteristics (ROC).^64^

- *Dimensionality reduction*. Since the preprocessed spectra were of high dimensionality, classification analysis on these data directly can be computationally expensive. Principal component analysis (PCA),^65^ as a widely used method for dimensionality reduction and feature extraction, was adopted to extract crucial information, that is, *features*, from the spectra data. For a n×p data matrix,

(1) $$X=\left[x_{1},x_{2},\ldots ,x_{p}\right],x_{i}={\left[x_{1i},x_{2i},\ldots ,x_{\mathrm{ni}}\right]}^{T},i=1,\ldots ,p,$$

a row vector in X belongs to $R^{p}$. PCA uses singular value decomposition (SVD)^66^ to extract l principal components (PCs) from X, with l<p. Each PC consists of element

(2) $$\begin{array}{c} t_{\mathrm{kj}}=\left[x_{j1},x_{j2},\ldots ,x_{\mathrm{jp}}\right]\cdot w_{k},j=1,2,\ldots ,n,k=1,2,\ldots ,l, \end{array}$$

where $w_{k}$ is the *weights* extracted by SVD that map each row of with X to a PC. The extracted PCs are ranked based on the percentage of data variance they explain. For example, the 1st PC explains the highest percentage of data variance among all the PCs; the 2nd PC explains the 2nd largest percentage of data variance, so on and so forth. These PCs are then the features to be used in subsequent classification analysis. In this study, R software was used for PCA implementation.

- *Feature selection*. The PCA reduces the high dimensionality of the Raman spectra (1008 independent variables from 648 cm^−1^ to 1747 cm^−1^) to a few PCs. To determine the optimal number of PCs for following machine learning models, *Quadratic Discriminant Analysis (QDA)* was performed with leave‐one‐out cross‐validation method.^67^ QDA is a classic type of binary classifier, which assumes normally distributed classes with unequal covariance. The two classes respectively follow $F_{0}=N\left(\mu_{0},\sum_{0}\right)$ and $F_{1}=N\left(\mu_{1},\sum_{1}\right)$. For a feature vector $t_{j}=\left[t_{j1},t_{j2},\ldots ,t_{\mathrm{jl}}\right]$, QDA predicts the *likelihood ratio* of the two classes,

(3) $$\begin{array}{c} L_{j}=\frac{{\sqrt{2\pi \left|\sum_{1}\right|}}^{-1}\exp\left(-\frac{1}{2}{\left(t_{j}-\mu_{1}\right)}^{T}\sum_{1}\left(t_{j}-\mu_{1}\right)\right)}{{\sqrt{2\pi \left|\sum_{0}\right|}}^{-1}\exp\left(-\frac{1}{2}{\left(t_{j}-\mu_{0}\right)}^{T}\sum_{1}\left(t_{j}-\mu_{0}\right)\right)},j=1,2,\ldots ,n \end{array}$$

Given a threshold of discrimination, T, if $L_{j}<T$, the $t_{j}$ is assigned class 0. Otherwise, it is assigned class 1. As shown in Figure S4, we compared the QDA classification accuracy for varying number of top PCs, and 15 PCs were chosen (accounts for almost 91.2% of variations in the data) for a good balance between high accuracy and small number of features. Thus, these 15 PCs are used in all following ML models.

- *Classification analysis*. Four other supervised learning classification methods, SVM, KNN, ANN and XGB were also performed by R software on these 15 PCs which served as input variables. The 90% SERS data set of sEVs were used to train the models. The remaining 10% spectral data were used to predict the CAD stages through the models.
- *Analysis of diagnostic capability*. To gain insights into the diagnostic capability of the proposed method, we adopted *receiver operating characteristic (ROC)* curves to analyze the classifiers' sensitivity and specificity. The conventional ROC is a graphic representation of the diagnostic capability for a binary classification model. It visualizes the model's *true positive rate* (TPR) against the *false positive rate* (FPR) as the discriminant threshold varies.
  TPR: rate of correctly predicting class 1 (TPR = sensitivity);
  FPR: rate of falsely predicting class 1 (FPR = 1 − specificity);

(4) $$\begin{array}{c} \mathrm{TPR}=\frac{\mathrm{TP}}{\mathrm{TP}+\mathrm{FN}};\mathrm{FPR}=\frac{\mathrm{FP}}{\mathrm{FP}+\mathrm{TN}} \end{array}$$

For a complete ROC curve, the area under curve (AUC) represents the capability of accurately predicting the positive cases, which is the larger the better. In our study, there are four classes. To enable the analysis with ROC curves, we counted the representation stage as positive response and other three stages as the negative response for calculation of Sensitivity_acc_ and Specificity_acc_ in ROC curves. However, in other conditions, Sensitivity and Specificity for the five ML models were calculated by counting HC group as negative and other three CAD stages as positive.

### 4.6 Statistical analysis

The mean size of small extracellular vesicles is expressed as means ± SD. The statistical differences among multiple groups were analyzed by ANOVA. The *p* values are shown in the figures as *p < 0.05, which are considered to be statistically significant.

## AUTHOR CONTRIBUTIONS

**Xi Huang:** Data curation (equal); formal analysis (equal); investigation (equal); methodology (equal); validation (equal); writing – original draft (equal). **Bo Liu:** Data curation (equal); formal analysis (equal); investigation (equal); methodology (equal); validation (equal); writing – original draft (equal). **Shenghan Guo:** Data curation (supporting); formal analysis (equal); software (equal). **Weihong Guo:** Conceptualization (equal); data curation (supporting); formal analysis (equal); software (equal). **Ke Liao:** Data curation (supporting). **Guoku Hu:** Data curation (supporting). **Wen Shi:** Data curation (supporting); methodology (supporting). **Mitchell Kuss:** Data curation (supporting); methodology (supporting). **Michael Duryee:** Resources (equal); writing – original draft (supporting). **Daniel Anderson:** Conceptualization (equal); funding acquisition (equal); supervision (equal); writing – review and editing (equal). **Yongfeng Lu:** Conceptualization (equal); funding acquisition (equal); supervision (equal); writing – review and editing (equal). **Bin Duan:** Conceptualization (equal); funding acquisition (equal); project administration (lead); supervision (equal); writing – review and editing (equal).

### PEER REVIEW

The peer review history for this article is available at https://publons.com/publon/10.1002/btm2.10420.

## Supporting information


**Figure S1** All processed Raman spectra of health control (HC) group and three CAD stages, SP, NSTEMI, and STEMI. The variations in spectra were significantly notable even within the same CAD stage.
**Figure S2** The plot of overall accuracy vs PC numbers to determine the optimized number of PCs used for all machine learning models.
**Figure S3** Raman data set of health control (HC) group and three CAD stages, SP, NSTEMI, and STEMI.
**Figure S4** Typical raw Raman data obtained from plasma samples of patient #8 (before data preprocessing). Black: spectrum without sEVs (only with PBS buffer). Blue: spectrum with high PBS peak and high background level (Removed). Red: spectrum with low PBS peak and low background level. (Kept for following machine learning models)
**Table S1** Averaged confusion matrix from the 50 round tests
**Table S2** Final coding and demographics of samples

## Data Availability

The data that support the findings of this study are available from the corresponding author upon reasonable request.
