# Supplementary material for: SERS spectroscopy with machine learning to analyze human plasma derived sEVs for coronary artery disease diagnosis and prognosis
Source: Bioeng Transl Med. 2022 Oct 5;8(2):e10420. doi: 10.1002/btm2.10420 (PMC10013764; doi:10.1002/btm2.10420)
Supplement: Supplementary file 1 — Figure S1 All processed Raman spectra of health control (HC) group and three CAD stages, SP, NSTEMI, and STEMI. The variations in spectra were significantly notable even within the same CAD stage. Figure S2 The plot of overall accuracy vs PC numbers to determine the optimized number of PCs used for all machine learning models. Figure S3 Raman data set of health control (HC) group and three CAD stages, SP, NSTEMI, and STEMI. Figure S4 Typical raw Raman data obtained from plasma samples of patient #8 (before data preprocessing). Black: spectrum without sEVs (only with PBS buffer). Blue: spectrum with high PBS peak and high background level (Removed). Red: spectrum with low PBS peak and low background level. (Kept for following machine learning models) Table S1 Averaged confusion matrix from the 50 round tests Table S2 Final coding and demographics of samples [file BTM2-8-e10420-s001.docx]

**Supporting Information**

**SERS spectroscopy with machine learning to analyze human plasma derived sEVs for coronary artery disease diagnosis and prognosis**

Xi Huang ^a,^ ^1^, Bo Liu ^b,c 1^, Shenghan Guo ^d,i^, Weihong Guo ^d^, Ke Liao ^e^, Guoku Hu ^e^, Wen Shi ^b,c^, Mitchell Kuss ^b,c^, Michael J. Duryee ^f^, Daniel R. Anderson ^c,^ *, Yongfeng Lu ^a,^ *, Bin Duan ^b,g,h^ *

^a^Department of Electrical and Computer Engineering, University of Nebraska Lincoln, Lincoln, NE, USA

^b^Mary & Dick Holland Regenerative Medicine Program, University of Nebraska Medical Center, Omaha, NE, USA

^c^Division of Cardiovascular Medicine, Department of Internal Medicine, University of Nebraska Medical Center, Omaha, NE, USA

^d^Department of Industrial and Systems Engineering, Rutgers, The State University of New Jersey, Piscataway, NJ, USA

^e^Department of Pharmacology and Experimental Neuroscience, University of Nebraska Medical Center, Omaha, NE, USA

^f^Division of Rheumatology, Department of Internal Medicine, University of Nebraska Medical Center, Omaha, NE, USA

^g^Department of Surgery, College of Medicine, University of Nebraska Medical Center, Omaha, NE, USA

^h^Department of Mechanical and Materials Engineering, University of Nebraska-Lincoln, Lincoln, NE, USA

^i^School of Manufacturing Systems and Networks, Arizona State University, Mesa, AZ, USA

^1^Equally contributed authors

^*^Corresponding authors:

Daniel Anderson Tel: + 1 (402) 5598129, E-mail address: [danderso@unmc.edu](mailto:danderso@unmc.edu)

Yongfeng Lu Tel: + 1 (402) 4728323, E-mail address: [ylu2@unl.edu](mailto:ylu2@unl.edu)

Bin Duan Tel: + 1 (402) 5599637, E-mail address: [bin.duan@unmc.edu](mailto:bin.duan@unmc.edu)


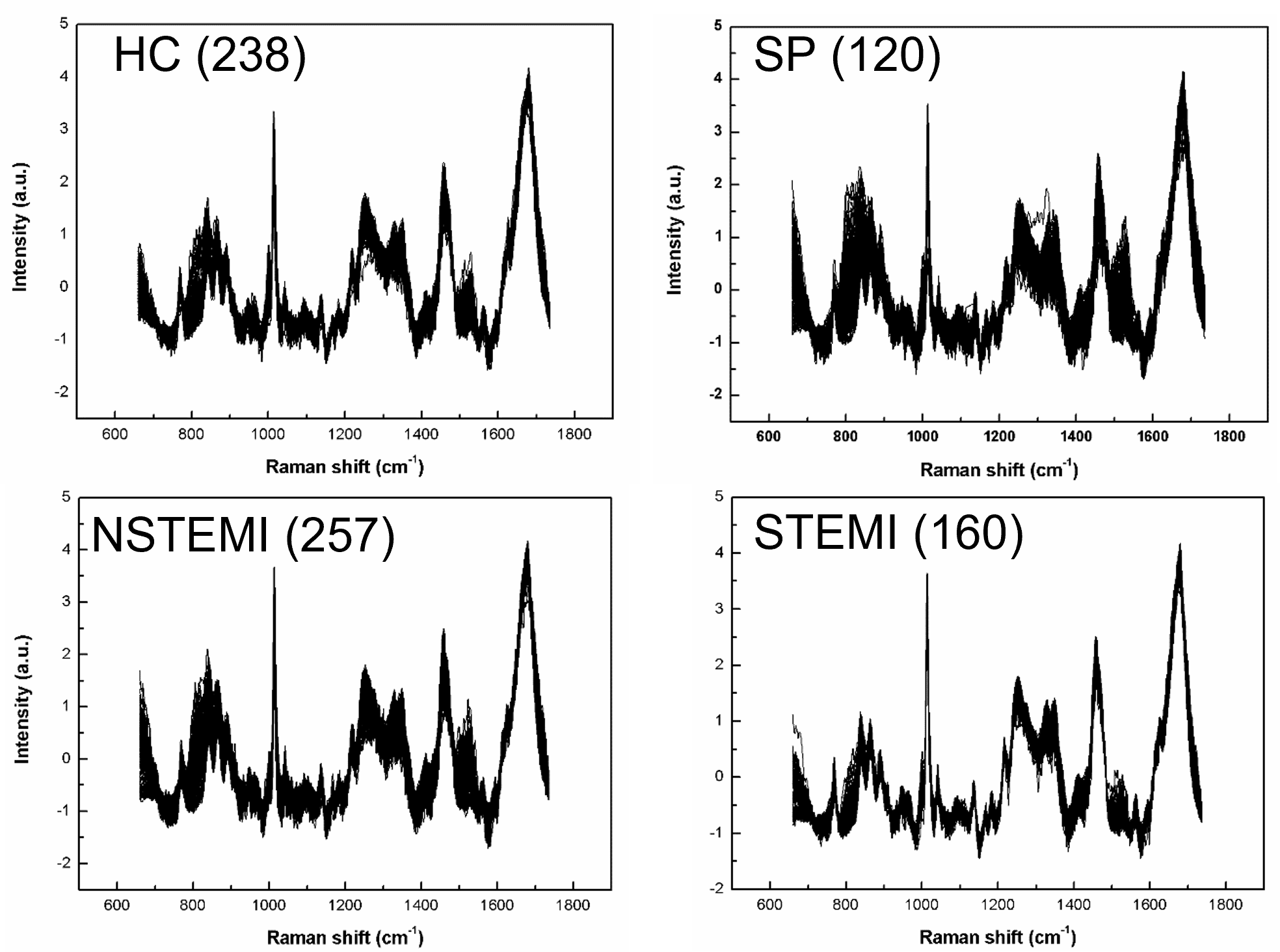


**Figure S1**. All processed Raman spectra of health control (HC) group and three CAD stages, SP, NSTEMI, and STEMI. The variations in spectra were significantly notable even within the same CAD stage.


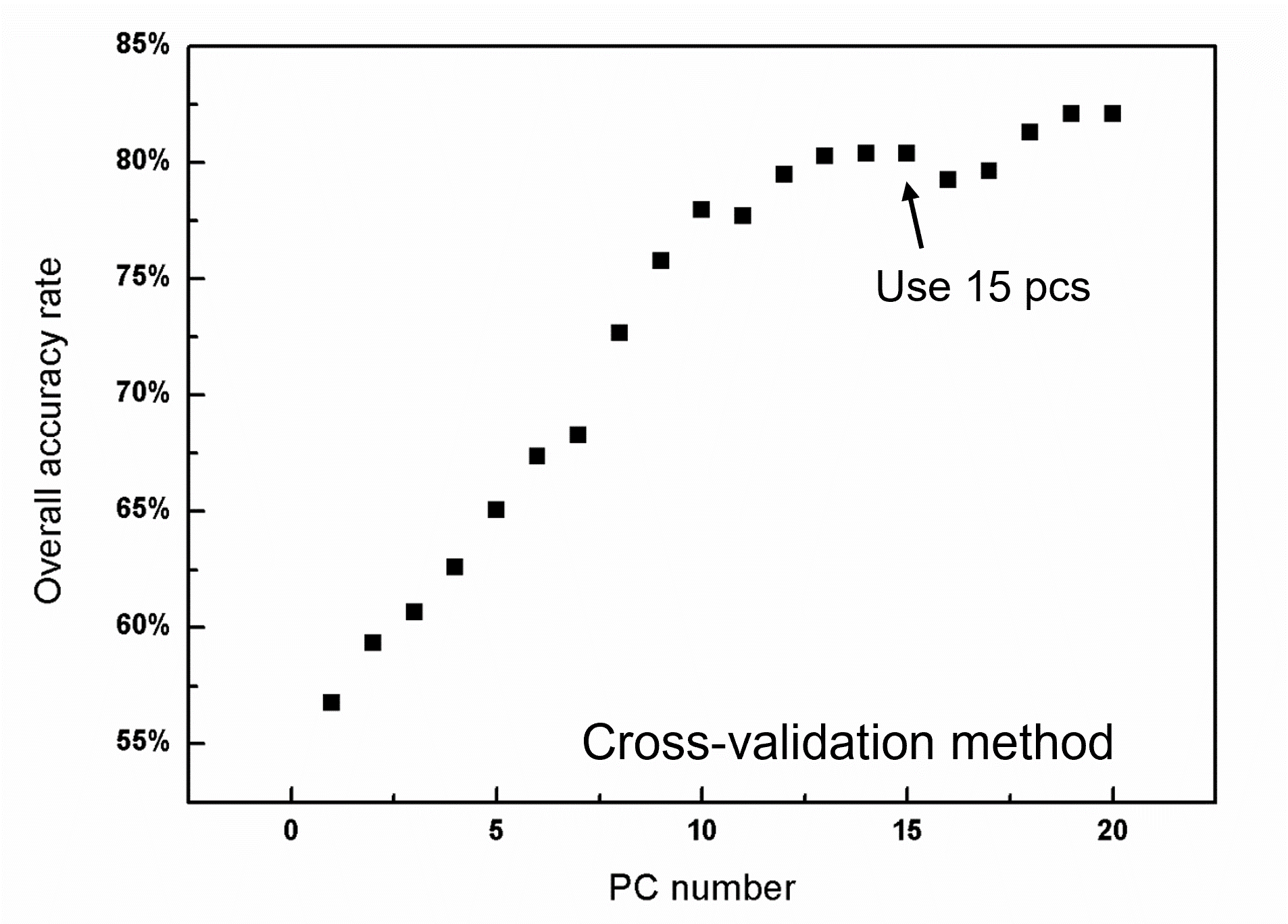


**Figure S2**. The plot of overall accuracy vs PC numbers to determine the optimized number of PCs used for all machine learning models.


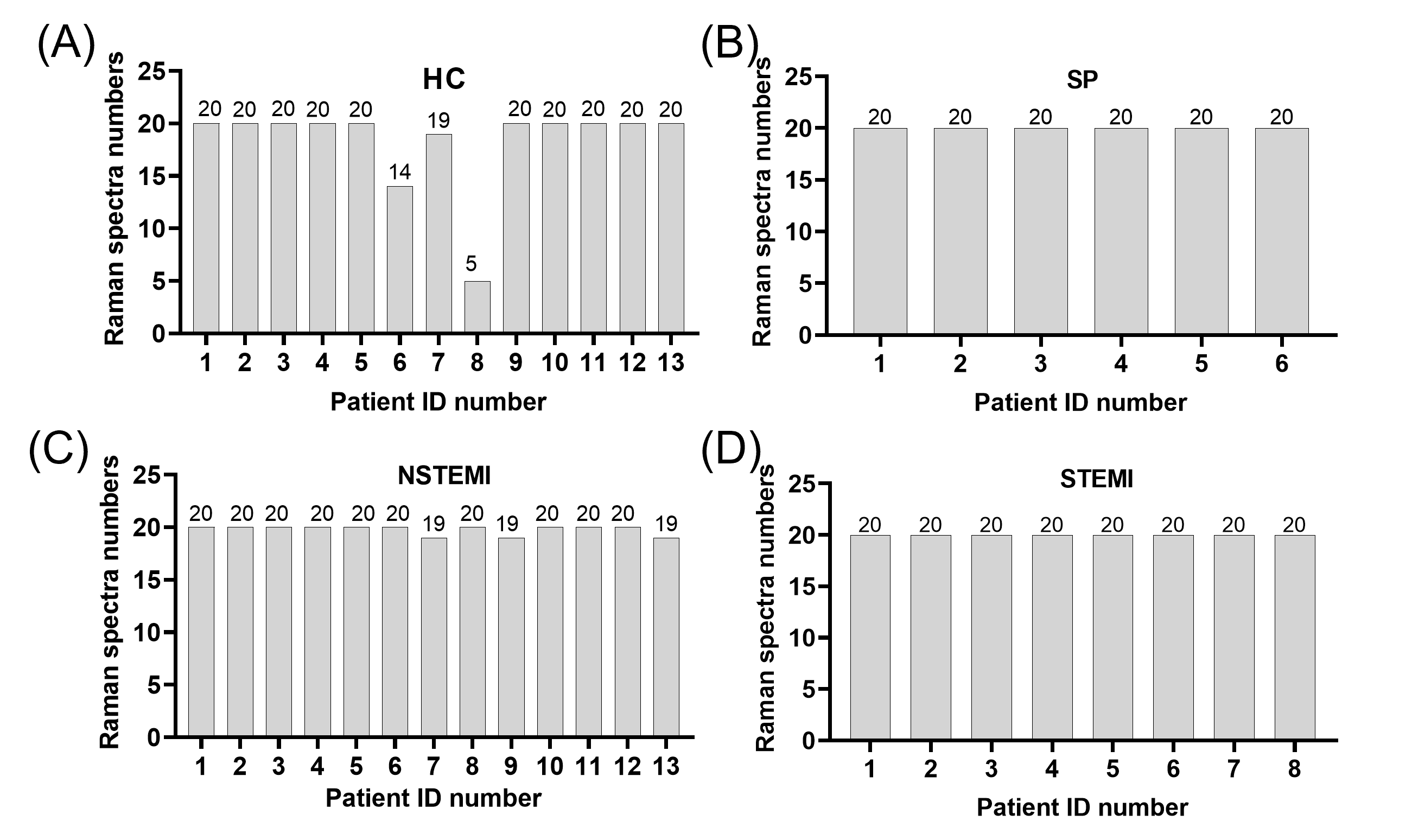


**Figure S3**. Raman dataset of health control (HC) group and three CAD stages, SP, NSTEMI, and STEMI.


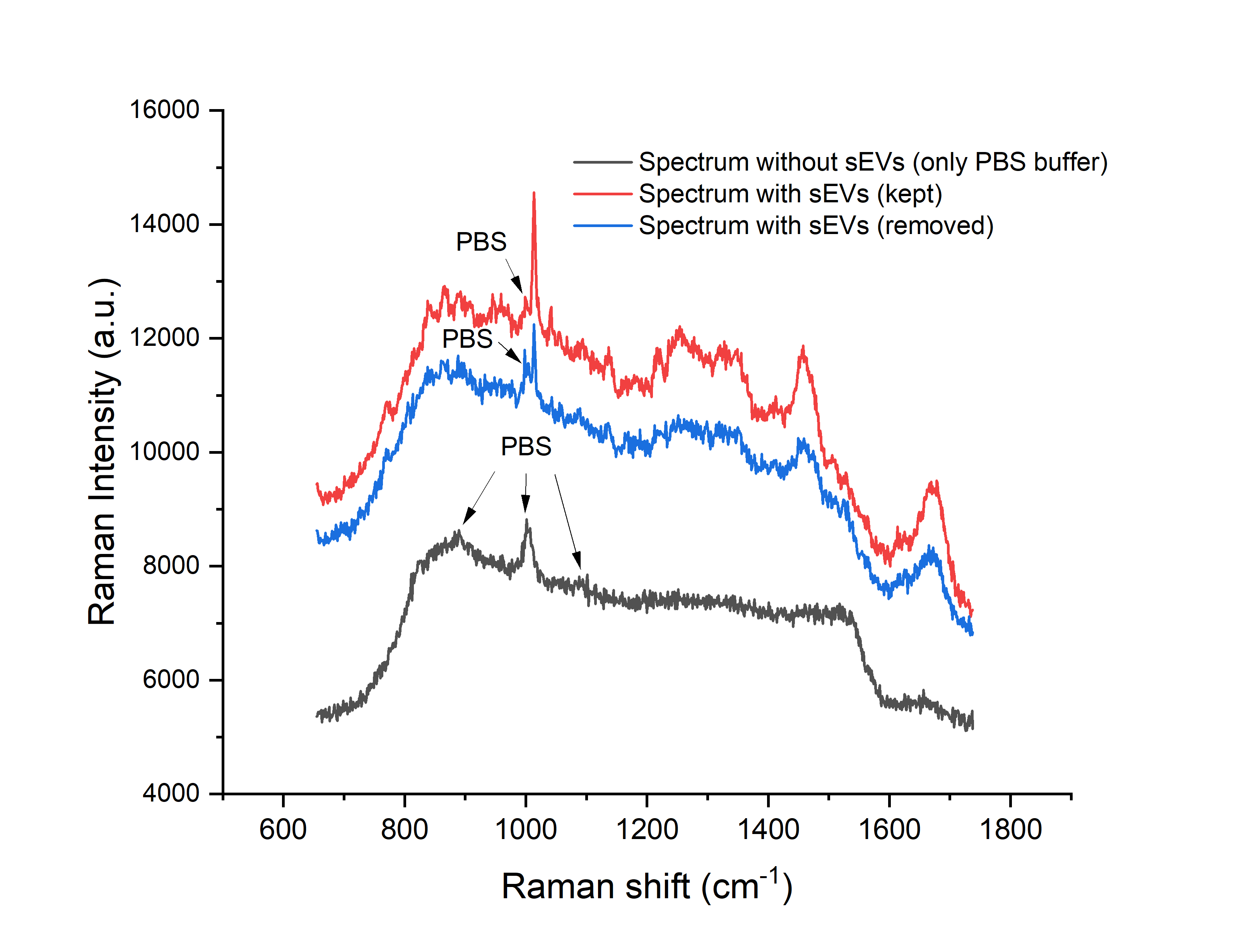


**Figure S4**. Typical raw Raman data obtained from plasma samples of patient #8 (before data preprocessing). Black: spectrum without sEVs (only with PBS buffer). Blue: spectrum with high PBS peak and high background level (Removed). Red: spectrum with low PBS peak and low background level. (Kept for following machine learning models)

**Table S1**. Averaged confusion matrix from the 50 round tests

| QDA | Classified as HC | Classified as SP | Classified as NSTEMI | Classified as STEMI |
| --- | --- | --- | --- | --- |
| HC | 74.7% | 4.3% | 8.2% | 12.7% |
| SP | 0.1% | 88.7% | 3.5% | 7.6% |
| NSTEMI | 7.0% | 0.0% | 78.0% | 14.9% |
| STEMI | 0.6% | 13.5% | 1.2% | 84.7% |
| SVM | Classified as HC | Classified as SP | Classified as NSTEMI | Classified as STEMI |
| HC | 95.7% | 0.1% | 2.4% | 1.9% |
| SP | 1.7% | 85.9% | 1.8% | 10.6% |
| NSTEMI | 4.4% | 0.5% | 94.4% | 0.7% |
| STEMI | 1.3% | 8.7% | 1.5% | 88.5% |
| KNN | Classified as HC | Classified as SP | Classified as NSTEMI | Classified as STEMI |
| HC | 91.6% | 0.6% | 4.6% | 3.2% |
| SP | 6.4% | 79.0% | 3.5% | 11.1% |
| NSTEMI | 7.0% | 0.9% | 91.0% | 1.2% |
| STEMI | 4.7% | 7.0% | 1.5% | 86.8% |
| ANN | Classified as HC | Classified as SP | Classified as NSTEMI | Classified as STEMI |
| HC | 88.5% | 0.0% | 2.2% | 9.2% |
| SP | 0.2% | 86.4% | 7.8% | 5.6% |
| NSTEMI | 9.0% | 3.0% | 82.8% | 5.1% |
| STEMI | 1.4% | 0.6% | 8.9% | 89.1% |
| XGB | Classified as HC | Classified as SP | Classified as NSTEMI | Classified as STEMI |
| HC | 82.2% | 0.8% | 12.4% | 4.5% |
| SP | 3.5% | 79.0% | 2.6% | 14.9% |
| NSTEMI | 13.5% | 0.5% | 84.2% | 1.8% |
| STEMI | 5.2% | 6.6% | 4.0% | 84.2% |

**Table S2**. Final coding and demographics of samples

| Disease groups *^a^* | Lab ID Number | SERS test times *^b^* | Bank ID | Gender *^c^* | Age | Race | NTA test *^d^* |
| --- | --- | --- | --- | --- | --- | --- | --- |
|  | 1 | 20 | CVB 014 | F | 52 | Black | - |
|  | 2 | 20 | CVB 018 | F | 47 | black | + |
|  | 3 | 20 | CVB 019 | M | 53 | Black | - |
|  | 4 | 20 | CVB 020 | F | 28 | Black | - |
|  | 5 | 20 | CVB 021 | M | 48 | White | - |
| HC | 6 | 14 | CVB 024 | F | 64 | White | + |
|  | 7 | 19 | CVB 025 | M | 67 | American Indian | + |
|  | 8 | 5 | CVB 026 | F | 55 | White | + |
|  | 9 | 20 | CVB 028 | M | 41 | Black | + |
|  | 10 | 20 | CVB 030 | F | 57 | White | + |
|  | 11 | 20 | CVB 031 | M | 57 | White | - |
|  | 12 | 20 | CVB 032 | M | 44 | White | - |
|  | 13 | 20 | CVB 038 | M | 45 | White | + |
|  | 14 | - | CVB 011 | F | 58 | White | + |
|  | 15 | - | CVB 013 | F | 53 | Black | + |
|  | 16 | - | CVB 033 | M | 44 | White | + |
|  | 17 | - | CVB 049 | M | 40 | Black | + |
|  | 1 | 20 | NCBR 192 | M | 61 | White | - |
|  | 2 | 20 | NCBR 193 | F | 63 | White | - |
| SP | 3 | 20 | NCBR 195 | F | 79 | White | - |
|  | 4 | 20 | NCBR 196 | M | 56 | White | + |
|  | 5 | 20 | NCBR 263 | M | 60 | White | + |
|  | 6 | 20 | NCBR 207 | F | 83 | White | - |
|  | 7 | - | NCBR 188 | F | 56 | White | + |
|  | 8 | - | NCBR 208 | F | 61 | White | + |
|  | 9 | - | NCBR 213 | M | 69 | Black | + |
|  | 10 | - | NCBR 219 | M | 69 | White | + |
|  | 11 | - | NCBR 232 | M | 41 | White | + |
|  | 12 | - | NCBR 248 | M | 63 | White | + |
|  | 13 | - | NCBR 255 | M | 71 | White | + |
|  | 14 | - | NCBR 274 | M | 75 | White | + |
|  | 15 | - | NCBR 275 | M | 69 | White | + |
|  | 1 | 20 | NCBR 190 | M | 85 | White | + |
|  | 2 | 20 | NCBR 197 | M | 76 | White | + |
|  | 3 | 20 | NCBR 198 | M | 70 | White | + |
|  | 4 | 20 | NCBR 239 | M | 56 | White | - |
|  | 5 | 20 | NCBR 258 | M | 79 | White | - |
|  | 6 | 20 | NCBR 273 | M | 66 | White | - |
|  | 7 | 19 | NCBR 276 | F | 78 | White | + |
| NSTEMI | 8 | 20 | NCBR 367 | M | 51 | Hispanic | + |
|  | 9 | 19 | NCBR 356 | M | 71 | White | + |
|  | 10 | 20 | NCBR 309 | M | 71 | White | + |
|  | 11 | 20 | NCBR 310 | F | 78 | White | - |
|  | 12 | 20 | NCBR 318 | M | 75 | White | + |
|  | 13 | 19 | NCBR 342 | M | 51 | White | + |
|  | 14 | - | NCBR 355 | M | 57 | White | + |
|  | 15 | - | NCBR 194 | F | 69 | White | + |
|  | 1 | 20 | NCBR 184 | M | 76 | White | - |
|  | 2 | 20 | NCBR 201 | M | 60 | White | + |
|  | 3 | 20 | NCBR 215 | M | 56 | White | - |
| STEMI | 4 | 20 | NCBR 217 | F | 69 | Hispanic | - |
|  | 5 | 20 | NCBR 230 | M | 49 | White | + |
|  | 6 | 20 | NCBR 243 | M | 55 | White | - |
|  | 7 | 20 | NCBR 251 | M | 81 | White | - |
|  | 8 | 20 | NCBR 358 | M | 52 | White | - |
|  | 9 | - | NCBR 279 | F | 61 | White | + |
|  | 10 | - | NCBR 250 | M | 58 | White | + |
|  | 11 | - | NCBR 272 | M | 71 | White | + |
|  | 12 | - | NCBR 303 | F | 42 | White | + |
|  | 13 | - | NCBR 312 | F | 48 | White | + |
|  | 14 | - | NCBR 319 | M | 72 | White | + |
|  | 15 | - | NCBR 343 | M | 70 | White | + |
|  | 16 | - | NCBR 278 | F | 61 | White | + |
|  | 17 | - | NCBR 373 | M | 65 | White | + |

*^a^* The groups HC, SP, NSTEMI and STEMI refer to Healthy control, Staple plaque, Non-ST-elevation myocardial infarction, and ST-elevation myocardial infarction, respectively.

*^b^* ‘-’refers to no application.

*^c^* F and M refer to female and male, respectively.

*^d^* ‘+’refers to application.
